# Validation of serum IGF-I as a biomarker to monitor the bioactivity of exogenous growth hormone agonists and antagonists in rabbits

**DOI:** 10.1242/dmm.016519

**Published:** 2014-09-19

**Authors:** Maximilian Bielohuby, Sayyed Hamid Zarkesh-Esfahani, Jenny Manolopoulou, Elisa Wirthgen, Katja Walpurgis, Mohaddeseh Toghiany Khorasgani, Zahra Sadat Aghili, Ian Robert Wilkinson, Andreas Hoeflich, Mario Thevis, Richard J. Ross, Martin Bidlingmaier

**Affiliations:** 1 Endocrine Research Unit, Medizinische Klinik und Poliklinik IV, Ludwig-Maximilians University, 80336 Munich, Germany.; 2 Department of Biology, Faculty of Sciences, University of Isfahan, Isfahan 81746-73695, Iran.; 3 Department of Immunology, Medical School, Isfahan University of Medical Sciences, Isfahan 81746-73461, Iran.; 4 The Department of Human Metabolism, The University of Sheffield, Sheffield S10 2JF, UK.; 5 Immunodiagnostic Systems, Boldon, Tyne and Wear NE35 9PD, UK.; 6 Ligandis GbR, Wilhelm-Stahl-Allee 2, 18196 Dummerstorf, Germany.; 7 German Sport University Cologne, Institute of Biochemisty/Center for Preventive Doping Research, 50933 Cologne, Germany.; 8 Leibniz Institute for Farm Animal Biology (FBN), Institute of Genome Biology, 18196 Dummerstorf, Germany.

**Keywords:** Pharmacodynamic marker, Acromegaly, Growth hormone deficiency, Animal model

## Abstract

The development of new growth hormone (GH) agonists and growth hormone antagonists (GHAs) requires animal models for pre-clinical testing. Ideally, the effects of treatment are monitored using the same pharmacodynamic marker that is later used in clinical practice. However, intact rodents are of limited value for this purpose because serum IGF-I, the most sensitive pharmacodynamic marker for the action of GH in humans, shows no response to treatment with recombinant human GH and there is little evidence for the effects of GHAs, except when administered at very high doses or when overexpressed. As an alternative, more suitable model, we explored pharmacodynamic markers of GH action in intact rabbits. We performed the first validation of an IGF-I assay for the analysis of rabbit serum and tested precision, sensitivity, linearity and recovery using an automated human IGF-I assay (IDS-iSYS). Furthermore, IGF-I was measured in rabbits of different strains, age groups and sexes, and we monitored IGF-I response to treatment with recombinant human GH or the GHA Pegvisomant. For a subset of samples, we used LC-MS/MS to measure IGF-I, and quantitative western ligand blot to analyze IGF-binding proteins (IGFBPs). Although recovery of recombinant rabbit IGF-I was only 50% in the human IGF-I assay, our results show that the sensitivity, precision (1.7–3.3% coefficient of variation) and linearity (90.4–105.6%) were excellent in rabbit samples. As expected, sex, age and genetic background were major determinants of IGF-I concentration in rabbits. IGF-I and IGFBP-2 levels increased after single and multiple injections of recombinant human GH (IGF-I: 286±22 versus 434±26 ng/ml; *P*<0.01) and were highly correlated (*P*<0.0001). Treatment with the GHA lowered IGF-I levels from the fourth injection onwards (*P*<0.01). In summary, we demonstrated that the IDS-iSYS IGF-I immunoassay can be used in rabbits. Similar to rodents, rabbits display variations in IGF-I depending on sex, age and genetic background. Unlike in rodents, the IGF-I response to treatment with recombinant human GH or a GHA closely mimics the pharmacodynamics seen in humans, suggesting that rabbits are a suitable new model to test human GH agonists and antagonists.

## INTRODUCTION

The development of pharmacological therapies to treat disorders of the growth hormone and insulin-like growth factor (GH-IGF) system allows better disease control and improves quality of life for the affected individuals. For medical treatment of GH excess (acromegaly), somatostatin analogs, dopamine agonists and a GH antagonist (GHA) are established in routine clinical practice (Katznelson et al., 2011). The synthesis of recombinant human GH and its approval for clinical use improved the treatment of individuals suffering from a GH deficiency (Kargi and Merriam, 2013). Pituitary secretion of GH is under the tight control of different hypothalamic releasing and inhibiting factors. Although GH itself is able to trigger biological responses in multiple target tissues, the most important ‘downstream’ target of GH is the liver, where GH stimulates IGF-I secretion through the activation of the hepatic GH receptor. In turn, IGF-I modifies GH secretion through a negative-feedback loop (Bielohuby et al., 2011a; Le Roith et al., 2001; Møller and Jørgensen, 2009). Therefore, in GH excess and GH insufficiency, the circulating concentration of IGF-I is considered to be the most important biomarker for diagnosis and monitoring of the disease (Clemmons, 2007; Keller et al., 2007). GH replacement therapy aims to normalize endogenous IGF-I concentrations, whereas effective treatment in individuals with acromegaly is reflected by decreasing IGF-I concentrations (Kargi and Merriam, 2013; Katznelson et al., 2011). Some studies also suggest that circulating IGF-binding proteins (IGFBPs) are sensitive markers for monitoring the disease and the treatment (Blum et al., 1993; Zapf et al., 1990). Because IGFBPs are determinants of serum IGF-I half-life, their measurement has been proposed to provide additional information in intervention studies (Metzger et al., 2011).

Despite undisputed beneficial effects of existing pharmaceutical options, novel potent substances are required because the available medical treatment of GH excess is not effective in all individuals, and treatment of GH deficiency still requires frequent injections. Furthermore, existing treatment options are accompanied by potential adverse effects, including increased hepatic enzymes, swelling, lipodystrophy at the injection sites, diarrhea and nausea (Péter et al., 2012; Petersenn et al., 2010; Sievers et al., 2009; van der Lely et al., 2012). Therefore, the development of novel drugs primarily aims to increase the potency of the substances, which ideally should lead to better disease control, fewer side effects and less frequent injections, or lower doses.

**TRANSLATIONAL IMPACT**

**Clinical issue**

Disorders of the growth hormone and insulin-like growth factor (GH-IGF) system lead to severe diseases in humans. For example, acromegaly is a life-threatening disease associated with GH-excess, mostly caused by a GH-producing tumor in the pituitary gland, whereas GH-deficiency, as seen in individuals with growth failure, is characterized by a lack of GH-secretion, which can be congenital or acquired in childhood or adult life. Existing pharmaceutical options for these disorders are not ideal because the available treatments for GH-excess are not effective in all affected individuals, whereas for GH-deficiency, more potent and long-acting preparations are required. Thus, novel human GH-agonists and GH antagonists (GHAs) displaying a higher efficacy and fewer side effects are needed. Ideally, the effects of a treatment with a novel substance are monitored in laboratory animals using a pharmacodynamic biomarker that is also available in clinical practice. In humans, serum IGF-I is the most sensitive biomarker for the diagnosis and treatment surveillance of GH-related diseases. Mice and rats are the most frequently used animal models for pre-clinical drug development, but are of limited value for this indication because they show a unique regulation of the GH-IGF system to that of humans. In this study, in order to identify a more suitable, non-rodent animal model, pharmacodynamic markers of GH action were investigated in rabbits.

**Results**

Owing to the lack of validated analytical platforms for the measurement of IGF-I concentrations in rabbits, an automated human immunoassay was tested for this purpose. The human IGF-I immunoassay exhibited excellent sensitivity, precision and linearity in rabbit samples. Similar to humans, sex, age and genetic background were major determinants of circulating IGF-I concentrations in rabbits. Single and multiple injections of recombinant human GH increased IGF-I concentrations, whereas treatment with a GHA lowered IGF-I in rabbits. Thus, in these animals, unlike in rodents, the IGF-I response to treatment with recombinant human GH or a GHA closely mimics the pharmacodynamics seen in humans.

**Implications and future directions:**

These results indicate that rabbits are a suitable new model to test human GH agonists and antagonists. For the first time, a validated immunoassay is now available to measure changes in circulating IGF-I together with a reference for detecting physiological IGF-I concentrations in rabbits. By using this automated high-throughput platform, translational research will be facilitated, because this system is already in use in many routine laboratories. The possibility to measure IGF-I in rabbits, together with the human-like behavior of IGF-I that was detected in response to recombinant human GH and GHA, will aid the development of novel therapeutics to treat pathological conditions of the GH-IGF system.

Animal models are indispensable for the development of novel drugs, and most commonly rodents are used to investigate the properties of novel compounds. However, for the testing of substances affecting the GH-IGF system, rodents only exhibit a limited suitability. We and others have previously reported that in intact mice and rats, IGF-I cannot be used as a reliable pharmacodynamic readout to monitor exogenous GH administration (Bielohuby et al., 2011b; Domené et al., 1993; Grönbladh et al., 2013). In contrast to the situation in most other mammals, including humans, IGF-I is not secreted at a higher rate in response to treatment with exogenous GH in intact rodents (Bielohuby et al., 2011b). To avoid this issue, hypophysectomized rodents are often used because the low endogenous IGF-I concentrations in these animals can be potently stimulated by treatment with exogenous GH. The major disadvantage of using hypophysectomized mice or rats for testing of GH agonists is that, in this artificial model, all pituitary hormones are lacking. Thereby, the normal mammalian physiology is largely disturbed, and it can not be predicted whether the absence of a physiological pituitary function itself affects experiments using GH agonists.

By contrast, GHA and somatostatin analogs have been shown to decrease circulating IGF-I concentrations in rodents (Courtland et al., 2011; Divisova et al., 2006; Grønbaek et al., 2002). Notably, GHAs per se (without previous treatments with exogenous GH) decrease circulating IGF-I levels in rodents with intact pituitary glands. However, it has been shown for the GHA Pegvisomant that, compared with individuals with acromegaly, mice need approximately 10- to 20-fold higher doses in order to induce a significant reduction of circulating IGF-I (Kopchick et al., 2014; Kopchick et al., 2002). With respect to somatostatin analogs, the limited suitability of rodents as a pharmacodynamic model is also evidenced by a recent study from Masyuk et al., in which the authors report that rats treated with the somatostatin analog Octreotide do not show a corresponding reduction in circulating IGF-I (Masyuk et al., 2013). These examples highlight that rodents display a unique regulation of the GH-IGF system to that of humans. Considering these shortcomings of rodents as a model to develop and test novel drugs in the field of GH-IGF-related disorders, we speculated that rabbits, which are also commonly used as a laboratory animal model, could be more suitable. This hypothesis was further driven by a study from Cota and colleagues who have previously generated a GH-overexpressing rabbit model (Costa et al., 1998). Interestingly, this model shares many of the clinical pathophysiological symptoms that are observed in individuals with acromegaly (e.g. growth of specific parts of the head) but are absent in GH-transgenic rodents (Costa et al., 1998). From an evolutionary point of view, rabbits taxonomically belong to the order of Lagomorpha, whereas mice and rats are classified as Rodentia. Although rodent models are obviously justified and display particular advantages for biomedical research, rabbits are also a widely used research model offering particular strengths over rodents (Daniel-Carlier et al., 2013; Flisikowska et al., 2011; Katter et al., 2013; Thakur et al., 2000). In addition, tremendous progress has been made in genetically modifying rabbits (Flisikowska et al., 2011; Katter et al., 2013), enabling in-depth analysis of genome function for future biomedical research. Thus, it can be assumed that the accurate measurement of IGF-I in rabbits is also of relevance for research beyond the treatment of GH-related disorders. For example, it has only recently been suggested in human studies that IGF-I is an important marker in malignant and metabolic diseases (Friedrich et al., 2012; Key et al., 2010; Rosskopf et al., 2011).

Until today, no commercially available IGF-I assay has been validated for the measurement of IGF-I in rabbit samples. Therefore, the first step in the investigation of the suitability of IGF-I as a pharmacodynamic marker of GH action in rabbits was to validate an assay intended for human IGF-I in rabbit samples through analyzing precision, sensitivity, linearity and recovery. For this purpose, we used the automated human IGF-I assay (iSYS, IDS), which recently has been extensively validated for human samples (Bidlingmaier et al., 2014; Friedrich et al., 2014). The second step was to analyze the physiological IGF-I and IGFBP concentrations in male and female rabbits of different genetic strains and age groups. Finally, we studied the suitability of rabbits as a new laboratory animal model for studies of the GH-IGF system by testing how endogenous IGF-I and IGFBP concentrations respond to treatment with either recombinant human GH or the GH antagonist Pegvisomant.

## RESULTS

### Assay validation

**Fig. 1.**
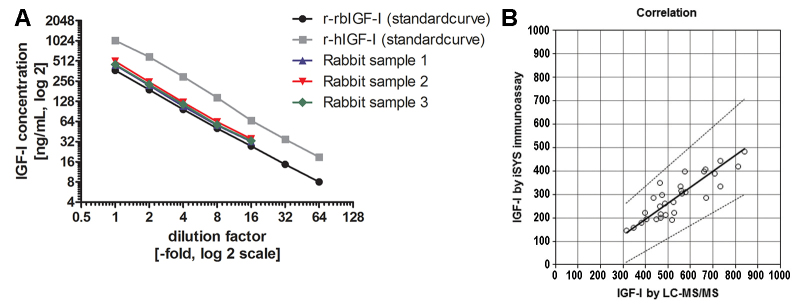
**Dilution linearity and correlation analysis with LC-MS/MS measurements.** (A) The dilution linearity of the assay with three different native rabbit serum samples in relation to recombinant rabbit IGF-I (r-rbIGF-I) and recombinant human (r-hIGF-I) standard curves. (B) The correlation of rabbit IGF-I measurements obtained using either the immunoassay or LC-MS/MS by using Passing–Bablock regression. Rabbit IGF-I concentrations were on average approximately twofold higher when measured by using LC-MS/MS (Spearman’s ρ, 0.813; *P*<0.0001; Passing–Bablock: slope 0.684 and intercept −78.3). The solid line shows the regression fit; the dotted lines show the 95% confidence limits of the regression fit.

A summary of the assay characteristics for the measurement of IGF-I in rabbit samples is displayed in Table 1. The intra-assay variability (precision) measured in rabbit serum displaying low, medium and high IGF-I concentrations was 2.55, 2.25 and 1.49%, respectively (Table 1; supplementary material Table S1). The inter-assay variability from six samples measured in five different assay runs was found to be between 1.7 and 3.3% with a mean variability of 2.4% (Table 1; supplementary material Table S2). As described previously for human samples (Bidlingmaier et al., 2014), for rabbit samples, a functional sensitivity for a concentration of IGF-I below 10 ng/ml (precisely 7.3 ng/ml) was confirmed (Table 1; supplementary material Table S3). Linearity of the assay for native rabbit serum across the range 19–585 ng/ml for the sample with a ‘high’ starting concentration (rabbit serum A) and 15–190 ng/ml for the sample with a ‘low’ starting concentration (rabbit serum B) was well within accepted margins with mean observed to expected ratios of 98.4% and 112%, respectively (Table 2). Overall, the ratio of the observed to expected values in dilution linearity experiments using rabbit samples with ‘high’ and ‘low’ starting concentrations (concentration range 15–585 ng/ml) was 104.4%. Analysis of the assay linearity using a small set of native human samples (human serum A and B) yielded similar results to those observed with native rabbit samples, and a mean percentage of the observed over expected values of 107.6% and 116.9% for human serum A and B, respectively, was calculated from measurement results (Table 2). A more detailed study investigating the linearity of human samples for measurement of IGF-I using this immunoassay is published elsewhere (Bidlingmaier et al., 2014). With respect to dilution linearity in native rabbit samples, serial dilutions of the three tested native rabbit samples paralleled the dilution of the recombinant standard material used (i.e. recombinant human IGF-I and recombinant rabbit IGF-I; Fig. 1).

**Table 1.**
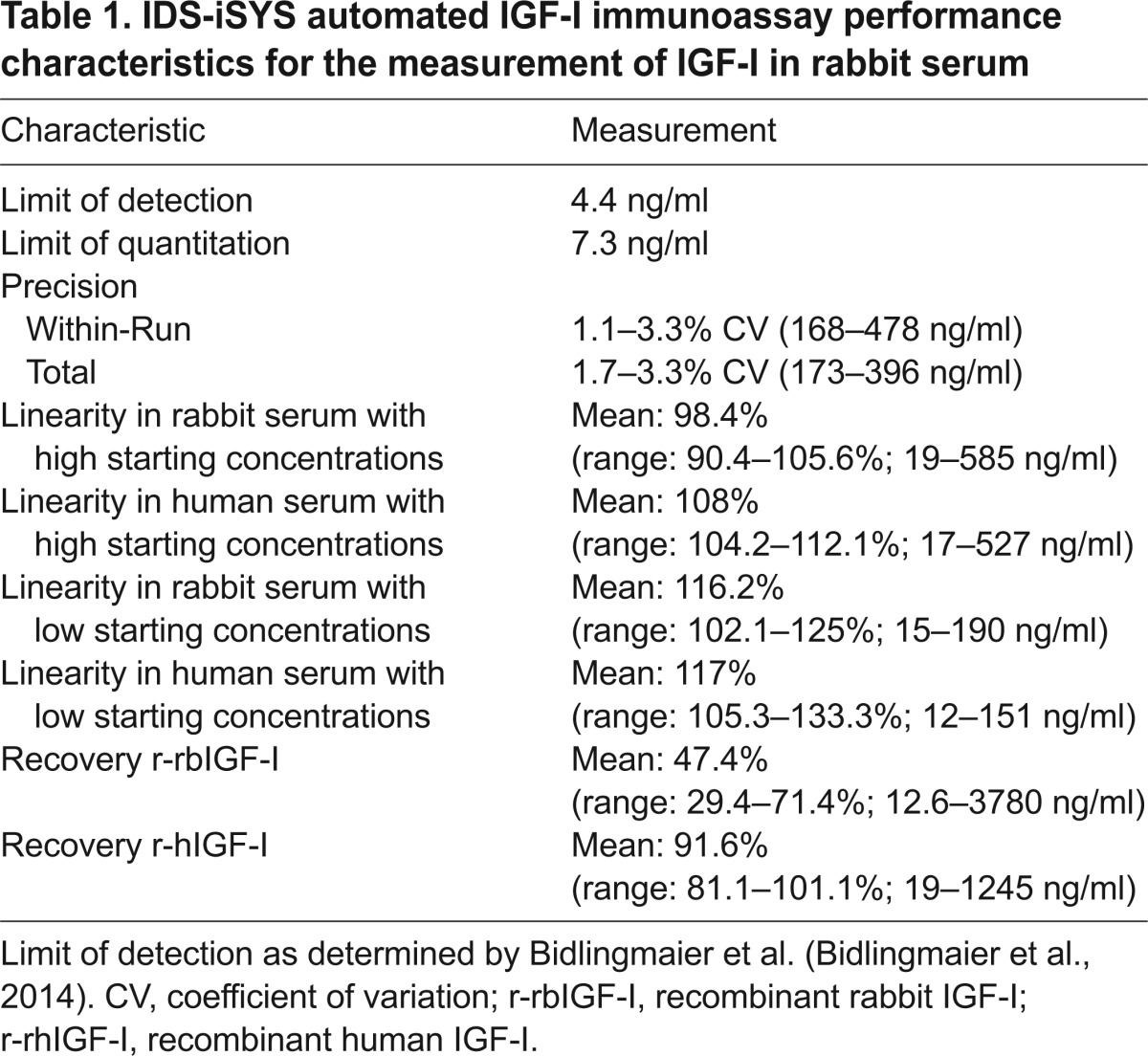
IDS-iSYS automated IGF-I immunoassay performance characteristics for the measurement of IGF-I in rabbit serum

**Table 2.**
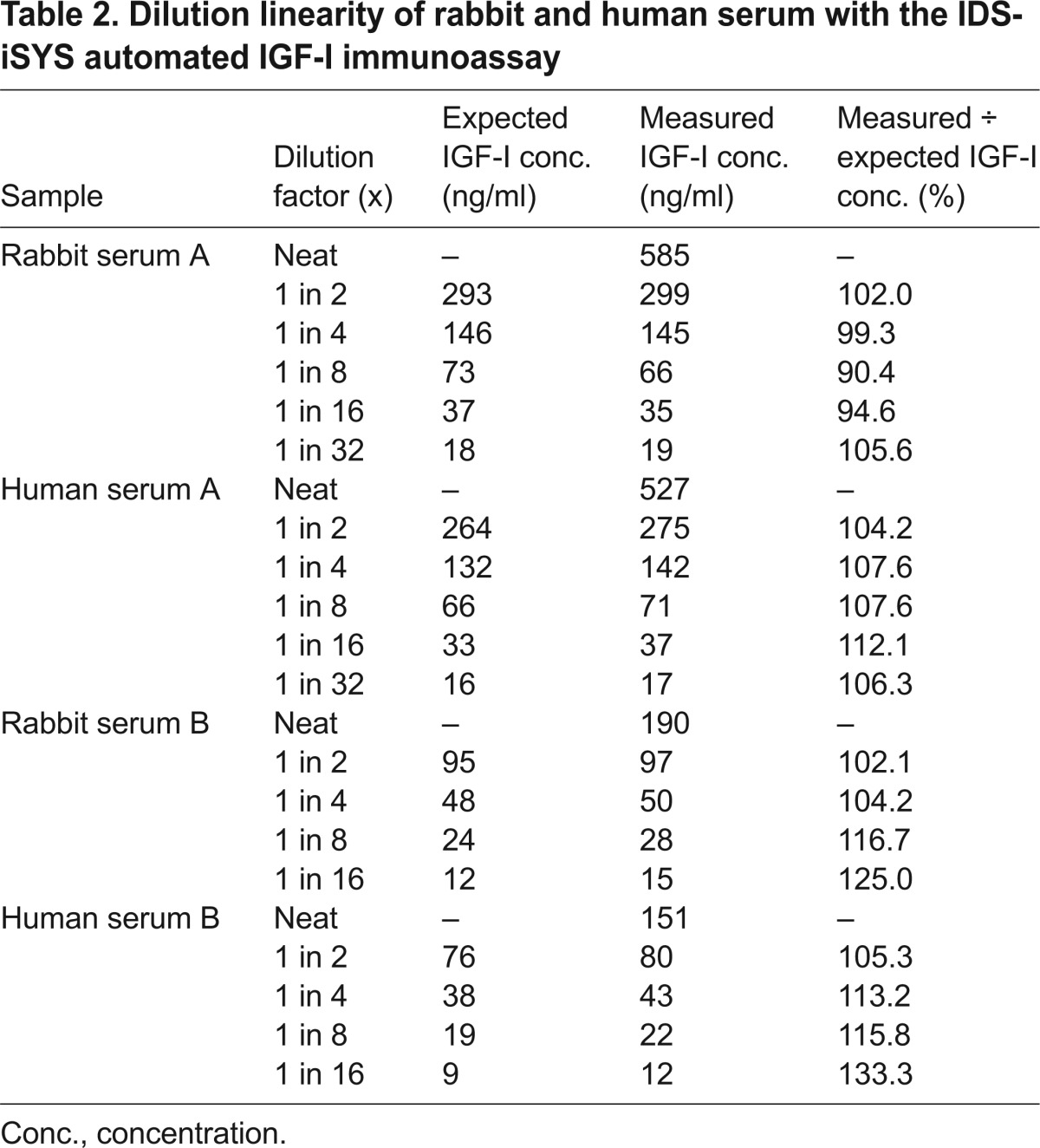
Dilution linearity of rabbit and human serum with the IDS-iSYS automated IGF-I immunoassay

Recovery of IGF-I concentrations was assessed by spiking assay buffer with either recombinant human IGF-I or recombinant rabbit IGF-I. As expected, samples spiked with recombinant human IGF-I (concentration range 19–1245 ng/ml) showed an excellent mean recovery rate of 91.6% (range 81.1–101.1%). By contrast, the mean recovery rate of recombinant rabbit IGF-I in assay buffer (concentration range 12.6–3780 ng/ml) was only 47.4% (range 29.4–71.4%; Table 3). Importantly, the lower recovery rate of recombinant rabbit IGF-I in this immunoassay (around 50%) also remained when using a different matrix and measuring native human or rabbit serum that had been spiked with recombinant rabbit IGF-I. For human serum spiked with recombinant rabbit IGF-I, the mean recovery rate upon measurement of IGF-I was 45.2% (range 40.4–53.2%), when using rabbit serum that had been spiked with recombinant rabbit IGF-I, a mean recovery rate of 53.9% (range 47.2–65.3%) was observed. Non-parametric correlation and regression analyses of the rabbit IGF-I measurements that had been made by using immunoassay and liquid chromatography tandem mass spectrometry (LC-MS/MS) revealed a good overall correlation of measurement results, but the rabbit IGF-I concentrations were on average 1.9-fold higher when measured by using LC-MS/MS (Spearman’s ρ, 0.813; *P*<0.0001; Passing–Bablock: slope 0.684 and intercept −78.3; Fig. 1B).

**Table 3.**
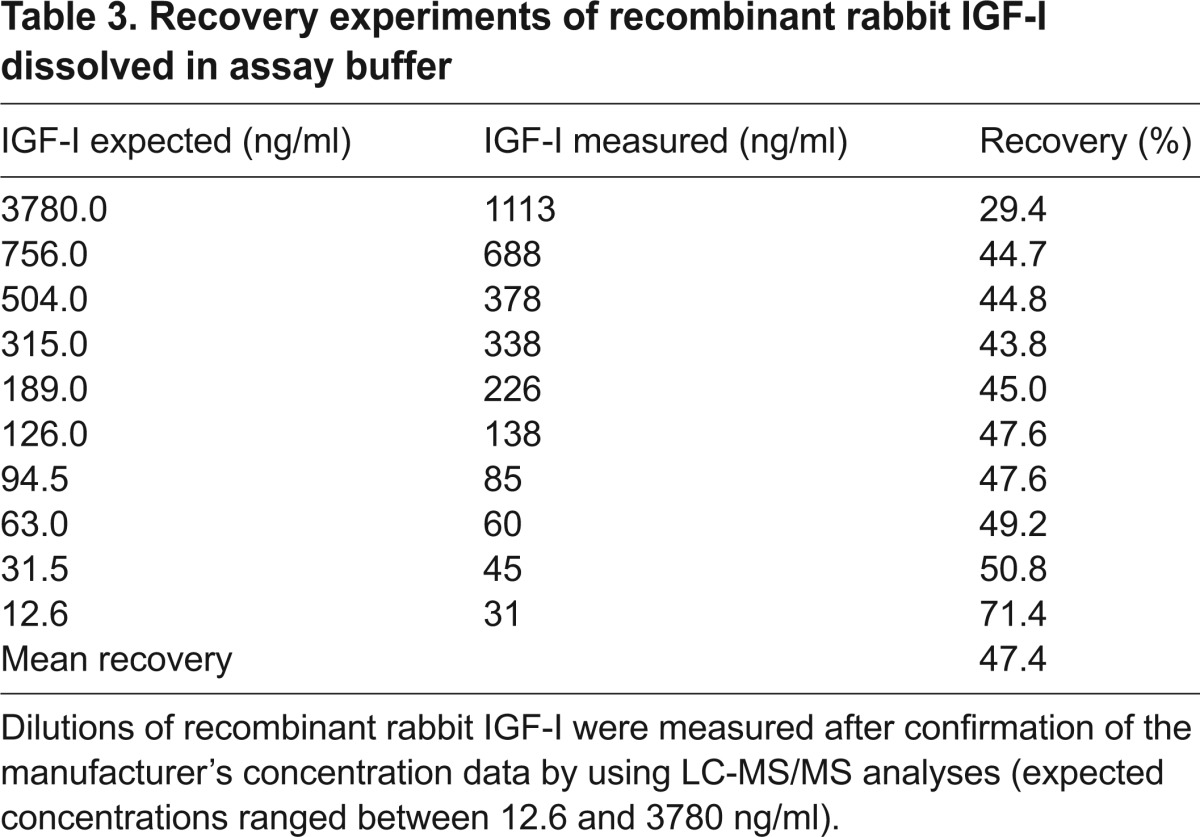
Recovery experiments of recombinant rabbit IGF-I dissolved in assay buffer

### Biological variability

**Fig. 2.**
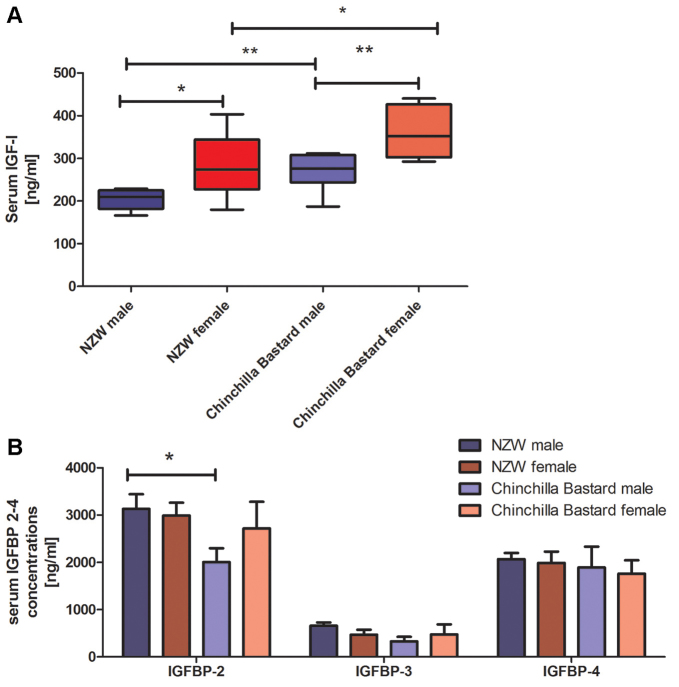
**Serum IGF-I and IGFBPs in rabbits.** (A) Serum IGF-I concentrations in male (blue) and female (red) rabbits from 13-week-old NZW and CB rabbits (males, *n*=7; females, *n*=8 for each strain). Data were analyzed by using non-parametric Mann–Whitney-U tests, *significant differences between male and female or between New Zealand White (NZW) and Chinchilla Bastard (CB) rabbits. Data are presented as box plots, center lines represent the median, boxes represent the middle 50% of data (interquartile range) and whiskers the 2.5–97.5 percentiles (*P<0.05, **P<0.01). (B) Analysis of serum IGFBPs by using quantitative western ligand blotting in male (blue) and female (red) rabbits from 13-week-old NZW and CB rabbits (males, *n*=7; females, *n*=8 for each strain). The analyses revealed that, in rabbits, the IGFBP-2 concentrations are quantitatively most abundant among the serum IGFBPs measured. With respect to sex and genetic background, IGFBP-2 concentrations were significantly (*P*<0.05) higher in 13-week-old NZW rabbits when compared with age-matched male CB rabbits. IGFBP-3 and IGFBP-4 were not significantly affected by sex or genetic background. Data are presented as means±s.e.m. (*P<0.05).

**Fig. 3.**
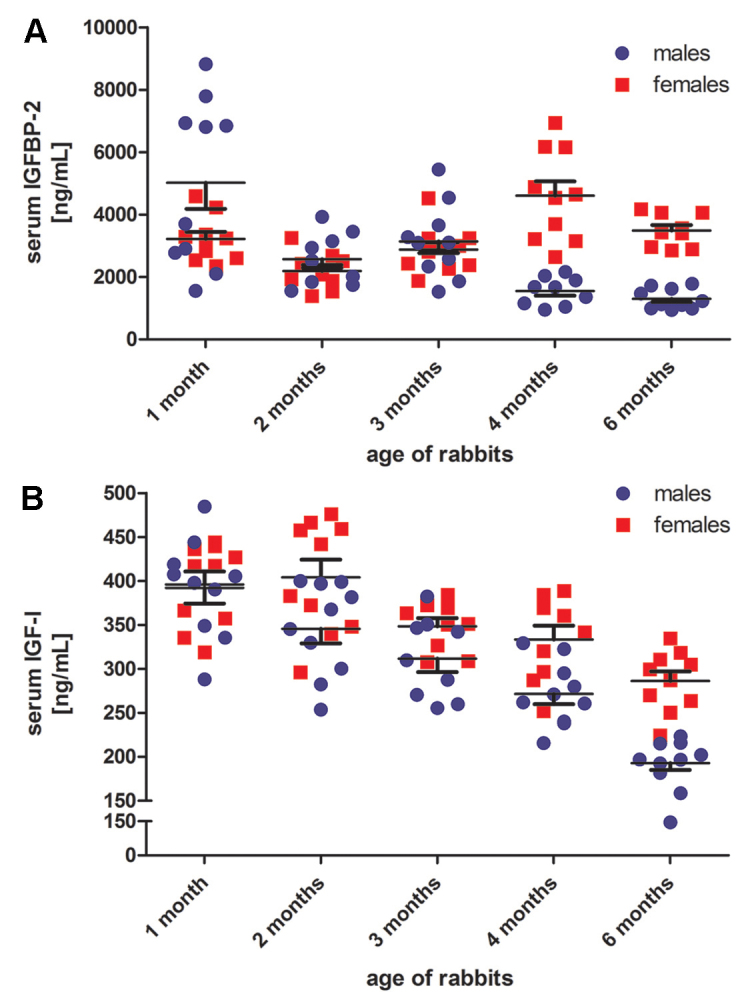
**Serum IGF-I and IGFBP-2 in 1- to 6-month-old rabbits.** (A) Serum IGFBP-2 concentrations (being the most abundant circulating IGFBP in rabbits) and (B) serum IGF-I concentrations in 1-, 2-, 3-, 4- and 6-month-old male (blue dots) and female (red dots) NZW rabbits (*n*=10 per sex and age group). Each dot represents one serum sample; the black lines indicate means±s.e.m. for each sex and age group, respectively (means with the s.e.m. bars ‘up’, females; means with the s.e.m. bars ‘down’, males). For illustrative purposes, the statistical comparison of age groups by using ANOVA is shown in the respective section of the results only.

The genetic background, sex and age of rabbits clearly affected circulating IGF-I concentrations. Analysis of samples from different rabbit strains showed significantly higher IGF-I concentrations in Chinchilla Bastard (CB) versus New Zealand White (NZW) rabbits (*P*<0.01 in males and *P*<0.05 in females; Fig. 2A). In both strains, female rabbits displayed significantly higher IGF-I concentrations (male NZW, 200.6±9.0 ng/ml; female NZW, 285.8±25.9 ng/ml, *P*<0.05; male CB, 265.7±16.3 ng/ml; female CB, 361.2±20.8 ng/ml, *P*<0.001; Fig. 2A). Also, the age of rabbits significantly affected IGF-I concentrations. Compared with 1-month-old rabbits, IGF-I concentrations in 3-, 4- and 6-month-old male rabbits were significantly lower (compared with the baseline concentrations at 1 month: 1 month versus 3 months *P*<0.05, versus 4 months and versus 6 months *P*<0.001, Fig. 3A). Similarly, IGF-I concentrations also declined in female rabbits with advancing age (females compared with the baseline concentrations at 1 month: 1 month versus 3 and 4 months *P*<0.05, versus 6 months *P*<0.001). The comparison of IGF-I concentrations in male and female rabbits throughout the different age groups revealed significantly higher IGF-I concentrations in female rabbits at the age of 2, 4 and 6 months (*P*<0.05, *P*<0.01 and *P*<0.001, respectively). No significant differences between sexes were found when comparing IGF-I concentrations from 1- and 3-month-old male and female rabbits (Fig. 3A). Results from IGF-I analyses using LC-MS/MS confirmed the age- and sex-specific differences in IGF-I concentrations, but the absolute concentrations were approximately twofold higher compared with the immunoassay measurements. By using LC-MS/MS, 6-month-old male NZW rabbits had significantly lower circulating IGF-I levels compared with 1-month-old male NZW rabbits and significantly higher IGF-I compared with 6-month-old female NZW rabbits (6-month-old male, 434.8±22.7 ng/ml versus 1-month-old male, 643.1±43.4 ng/ml, *P*<0.01 and versus 6-month-old female: 542.8±31.5 ng/ml, *P*<0.05).

### Circulating IGFBPs in rabbits of different sex, age and genetic background

Analysis of serum IGFBPs by using quantitative western ligand blotting revealed that, in rabbits, the IGFBP-2 concentrations were quantitatively most abundant among the serum IGFBPs that we measured (Fig. 2B). Overall, IGFBP-2 concentrations showed the highest correlation with serum IGF-I concentrations (as measured by using LC-MS/MS; Spearman’s ρ, 0:63, *P*<0.0001), followed by a moderate correlation of IGFBP-4 with serum IGF-I (Spearman’s ρ, 0:48, *P*<0.001). Circulating IGFBP-3 concentrations did not show a significant correlation with serum IGF-I. With respect to sex and genetic background, IGFBP-2 concentrations were significantly (*P*<0.05) higher in 13-week-old NZW rabbits when compared with age-matched male CB rabbits. IGFBP-3 and IGFBP-4 concentrations were not significantly affected by sex and genetic background. Fig. 3B displays serum IGFBP-2 concentrations in male and female NZW rabbits between 1 and 6 months of age. At the age of 4 and 6 months, serum IGFBP-2 concentrations were significantly higher in female rabbits compared with those of age-matched males (at 4 months, *P*<0.001; at 6 months, *P*<0.01; Fig. 3B). The other IGFBPs showed much lower variability across this period.

### Treatment of rabbits with a growth hormone antagonist

**Fig. 4.**
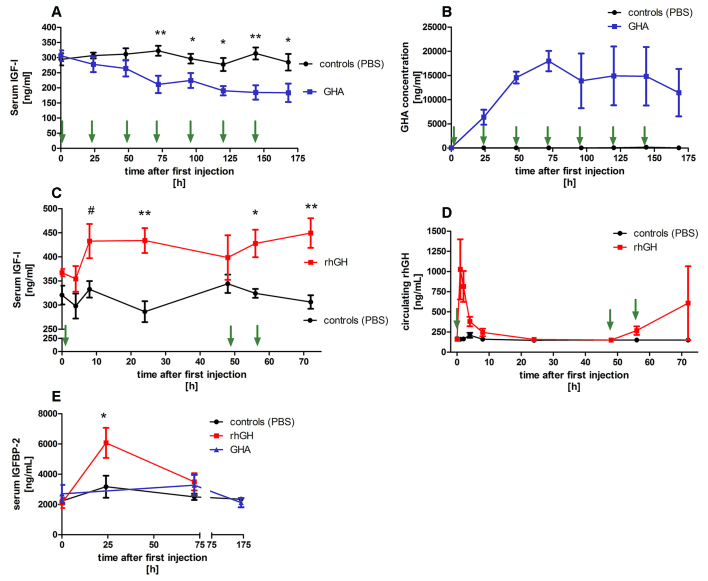
**Effect of treatment with recombinant GH or a GHA on serum IGF-I and IGFBP-2.** (A) Concentrations of serum IGF-I and (B) circulating concentrations of the growth hormone antagonist Pegvisomant (GHA) in GHA-treated rabbits. The green arrows indicate the injection time points (1 mg/kg of body weight). Controls received equal volumes of PBS. Serum samples were collected before injection at baseline (0 h) and 24, 48, 72, 96, 120, 144 and 168 h after the first GHA injection. Concentrations of (C) serum IGF-I and (D) circulating concentrations of recombinant human growth hormone (rhGH) in rabbits that had been treated with recombinant human GH. The green arrows indicate the injection time points (1 mg/kg of body weight). Controls received equal volumes of PBS. Serum samples were collected before injection at baseline (0 h) and 4, 8, 24, 48, 56 and 72 h after the first injection of recombinant human GH. (E) Concentrations of circulating IGFBP-2 in rabbits that had been treated with recombinant human GH (red line), GHA (blue line) or PBS (black line). Data were analysed by using two-way ANOVA with subsequent Bonferroni correction for multiple-comparison post-hoc tests. Data are presented as means±s.e.m. (^#^*P*=0.054, *P<0.05, **P<0.01).

**Fig. 5.**
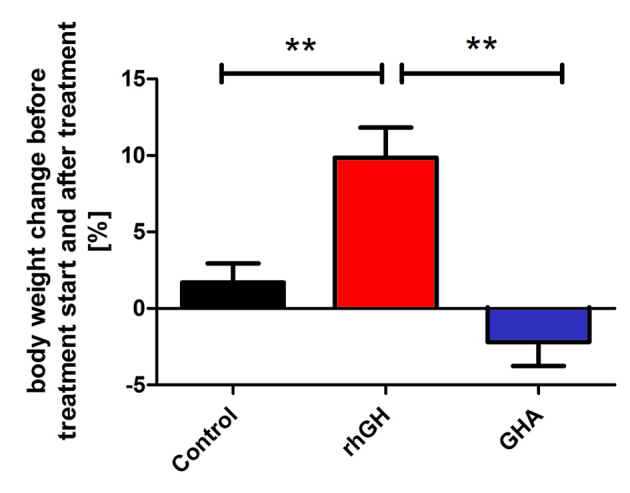
**Changes in the body weight of rabbits before the start of treatment with recombinant human GH or a growth hormone antagonist (GHA) and after termination of the treatment protocols.** Data were analysed by using ANOVA with subsequent Bonferroni correction for multiple-comparison post-hoc tests. Data are presented as means±s.e.m. (*P<0.05, **P<0.01).

During treatment with the GHA Pegvisomant, IGF-I concentrations in rabbits showed a continuous decline when compared with the baseline concentration. After the fourth injection (i.e. 72 h into the experiment), IGF-I concentrations were significantly (*P*<0.01) lower when compared with those of controls, which received injections of PBS. IGF-I concentrations also remained lower in GHA-treated rabbits when compared with controls at 96, 120, 144 and 168 h (i.e. after the fifth, sixth and seventh injection; Fig. 4A). Serum concentrations of Pegvisomant increased until the fourth injection to reach a maximum circulating concentration of 17990±2107 ng/ml. The GHA concentrations remained at this high level until the last injection was given and then declined again (Fig. 4B). As expected, the GHA was not detectable in the control group. Supplementary material Fig. 1 shows that after a single subcutaneous injection of the GHA, the serum GHA levels steadily increased to reach the highest GHA concentrations after 8 h. In comparison to recombinant human GH, the GHA was cleared much more slowly from the circulation (supplementary material Fig. S1). No effect of treatment with the GHA was observed on circulating concentrations of IGFBP-2, IGFBP-3 and IGFBP-4 concentrations (Fig. 4E; data not shown). Rabbits in the control group gained on average 1.7±1.2% body weight during the experiment, rabbits treated with the GHA displayed 2.2±1.6% lower body weight at the end of the experiment. However, the difference in body weight changes between controls and GHA-treated rabbits was not statistically significant (Fig. 5). Liver weights were not significantly different between control rabbits and rabbits that had been treated with the GHA (controls 76.6±5.3 g versus GHA 80.1±8.0 g).

### Treatment of rabbits with recombinant human growth hormone

In comparison to controls receiving PBS, even after only the first injection, serum IGF-I was significantly higher in rabbits that had been injected with recombinant human GH (versus controls after 8 h *P*=0.054, after 12 h *P*<0.01). IGF-I concentrations declined after the first injection with recombinant human GH, and 48 h after the first injection, IGF-I concentrations were no longer higher in rabbits that had been treated with recombinant human GH compared with controls. After the second and third injection of recombinant human GH (after 56 h and 72 h), IGF-I levels were significantly increased again (Fig. 4C). The combination of IGF-I concentrations at time points 24 h, 56 h and 72 h also resulted in significantly higher IGF-I concentrations (+42.8%) in the group that had been treated with recombinant human GH compared with those of controls (PBS 305.7±9.4 ng/ml versus recombinant human GH 436.7±15.1ng/ml; *P*<0.001; Fig. 4C). In contrast to GHA, recombinant human GH displayed a different pharmacodynamic behavior in the circulation. Peak concentrations were detected 8 h after an injection, with rapidly decreasing blood concentrations of recombinant human GH thereafter (Fig. 4D; supplementary material Fig. S1). IGFBP-2 concentrations in rabbits increased approximately threefold 24 h after being injected with recombinant human GH (*P*<0.001; Fig. 4E). IGFBP-3 and IGFBP-4 levels were not significantly affected by recombinant human GH (data not shown). With respect to changes in body weight, the recombinant-human-GH group showed the highest increases (+9.9±2.0%) in body weight when comparing body weights at the start of the treatment and after the last injection (Fig. 5). Compared with those of controls and rabbits treated with GHA, the body weight increases were significantly higher in rabbits that had been treated with recombinant human GH (*P*<0.01).

## DISCUSSION

We have demonstrated that in rabbits the measurement of IGF-I is a suitable option to monitor the pharmacodynamic effects of treatment with recombinant human GH and a GHA. Therefore, rabbits, in combination with the measurement of treatment-induced changes in circulating IGF-I, could easily be used as a new laboratory animal model to develop novel GH agonists and GH antagonists. Similar to humans and other mammals, rabbits also display a significant variability in circulating IGF-I depending on age, sex and genetic background. Furthermore, we have shown that the automated IDS-iSYS human IGF-I immunoassay can be used to reliably measure serum IGF-I concentrations in rabbits.

To our knowledge, this study is the first validation of a commercially available automated IGF-I immunoassay for the measurement of IGF-I in rabbits. We have decided to validate an existing automated IGF-I immunoassay for use with rabbits because the iSYS system is already used in many endocrine laboratories. Thereby, translational investigations are facilitated and the same analytical platform can be used for pre-clinical and clinical studies. Furthermore, the automated system allows for high-throughput analyses at reasonable costs, and the good performance of this system for measurement of IGF-I in human samples has only recently been published (Bidlingmaier et al., 2014). The low variability of measurement results during testing for linearity, and the intra- and inter-assay variability demonstrate that this assay can reliably measure IGF-I concentrations in rabbits. During the validation process, we confirmed a functional sensitivity of the assay for measurement of IGF-I concentrations below 10 ng/ml in rabbits, which allows us to measure even highly suppressed IGF-I concentrations and to accurately measure diluted samples in cases when the available volume is low. Overall, the validation of this assay revealed that the performance characteristics of the assay for measurement of IGF-I in rabbits are in line with the standards for human diagnostics outlined in the Clinical and Laboratory Standards Institute recommendations, as well as in a recent consensus statement (Clemmons, 2011; NCCLS, 2004).

However, the experiments using recombinant rabbit IGF-I, as well as the measurements made using LC-MS/MS, suggest a reduced affinity for rabbit IGF-I of one or both of the antibodies used in this human IGF-I assay. The biochemical structure of rabbit and human IGF-I is almost identical with the exception of a single amino acid exchange at the C-terminal fragment (UniProt database: exchange of serine to alanine in rabbit IGF-I at position 69). The capture antibody of the IDS-iSYS IGF-I assay binds to the N-terminus (Bidlingmaier et al., 2014) and thus should recognize human and rabbit IGF-I to an equal extent. Most likely, the acridinium-labeled antibody in the assay targets an epitope that includes the C-terminal part of the IGF-I molecule, or the epitope is conformationally changed by the one amino acid difference between both species. This might offer a possible explanation as to why the recovery of recombinant rabbit IGF-I was only around 50%. The assumption that the difference in the amino acid sequences also translates into a substantial difference in the 3D-structure of the IGF-I molecule is also supported by the observation of the reduced bioassay activity of the recombinant rabbit IGF-I tested in the human MCF7 cell line (see Materials and Methods). In summary, the true IGF-I concentrations in rabbits seem to be higher than those measured by using the immunoassay and, presumably, are comparable to the IGF-I concentrations observed in rodents (Bielohuby et al., 2013). Direct evidence for this statement derives from the IGF-I measurements made using LC-MS/MS, which clearly showed higher IGF-I concentrations in all samples compared to those obtained from the immunoassay. Owing to the limited number of samples analyzed using both methods, this study cannot provide a definite conversion factor for the immunoassay results. However, our data suggest that the true IGF-I concentrations in rabbits are on average approximately 1.9-fold higher than those measured by using the immunoassay.

Of note, we have also previously tried to analyze IGFBP-3 in rabbit samples using an assay running on the same automated platform (Friedrich et al., 2014). However, we were not able to detect IGFBP-3 in rabbit samples using the automated system. Therefore, we investigated IGFBP concentrations by western ligand blotting, using a novel approach for quantitative determination of IGFBPs independent of the species (Laeger et al., 2014; Metzger et al., 2011). In agreement with previously published data from other groups (Nason et al., 1996; Wolf et al., 1997), we confirmed that IGFBP-2 is the most abundant circulating IGFBP in rabbits. Exogenous GH stimulated an almost twofold increase of IGFBP-2 levels in rabbits, whereas, in humans, serum concentrations of IGFBP-2 are suppressed by the administration of GH (Bannink et al., 2006; Kassem et al., 1998; Katz et al., 1996). This not only reveals a general difference in the control of IGFBP-2 between humans and rabbits but identifies IGFBP-2 as a sensitive indicator of the actions of GH in rabbits. Importantly, IGFBP-2 concentrations showed a highly significant correlation with those of IGF-I in the serum of rabbits, which was much less pronounced for IGFBP-4 or even absent for IGFBP-3. This is in line with data from Wolf and colleagues who show higher IGFBP-2, but not IGFBP-3 and IGFBP-4, concentrations in rabbits overexpressing IGF-I (Wolf et al., 1997). So far, only a few reports on circulating IGFBP concentrations have been published for rabbits. One study reports a decline of all serum IGFBPs in early pregnancy compared with non-pregnant rabbits, as measured by using semi-quantitative western-ligand blotting, with a strong increase in IGFBP concentrations by mid-gestation until the late pregnancy (Nason et al., 1996). Interestingly, the intensities of the detected IGFBPs (IGFBP-2, IGFBP-3 and IGFBP-4) paralleled the changes in concentrations of serum IGF-I and IGF-II during pregnancy, suggesting that rabbits also display a molar ratio of approximately 1:1 for IGFs and IGFBPs (Nason et al., 1996). In our study, changes in serum IGFBP-2 reflected the biological differences of circulating IGF-I, as seen, for example, in the parallel increase of IGF-I and IGFBP-2 after recombinant human GH treatment or in 4- and 6-month-old female rabbits, which showed higher IGF-I and IGFBP-2 concentrations when compared to male rabbits of the same age. Our results highlight that IGF-I concentrations in rabbits are influenced by age, sex and genetic background. Although never systematically analyzed in rabbits before, this is not surprising, because these factors are known variables for IGF-I concentrations in mammals (Bidlingmaier et al., 2014; Le Roith et al., 2001). By contrast, owing to the fact that rabbits are widely used in biomedical research, it is surprising that only a few studies have investigated serum IGF-I and IGFBP concentrations in rabbits.

In 1984, D’Ercole et al. reported that they had developed and validated a radioimmunoassay (RIA) for the measurement of IGF-I in rabbit serum (relative to a human standard serum) (D’Ercole et al., 1984). The authors investigated IGF-I levels in pregnant and non-pregnant NZW rabbits. There were no significant differences between pregnant and non-pregnant rabbits, and the measured concentrations ranged between 4 and 7 U/ml. When employing a conversion factor of 150 ng/ml per 1 U/ml IGF-I (Festoff, 1995), our measurement results in NZW female rabbits were similar to those reported by D’Ercole and colleagues (D’Ercole et al., 1984). However, the RIA developed by D’Ercole et al. has not been validated with respect to linearity, precision or recovery and, to the best of our knowledge, no further studies measuring rabbit IGF-I with this assay have been published thereafter. Using the Nichols Research Institute human IGF-I RIA, which is no longer available, Turner et al. measured IGF-I in the serum of 3.5-month-old female Dutch-Belted rabbits to investigate whether oral fluoride supplementation affected skeletal growth factors (Turner et al., 1997). The authors of this study showed IGF-I concentrations between 66 and 92 ng/ml. Costa et al. generated transgenic rabbits overexpressing bovine GH (bGH) and measured IGF-I by using the human IGF-I RIA from Nichols (Costa et al., 1998). In their study, control rabbits had mean IGF-I concentrations of 149 ng/ml, the rabbits that overexpressed bGH showed approximately 4.5-fold higher circulating levels of IGF-I. Nason and co-workers systematically investigated serum IGF-I in NZW rabbits during the course of pregnancy by using the RIA (Nason et al., 1996). Non-pregnant rabbits showed mean IGF-I concentrations of 498 ng/ml, which peaked at day 21 of gestation, followed by a decline to 341 ng/ml by day 30. Other authors have only sporadically measured IGF-I in different rabbit model systems, reporting IGF-I concentrations between 87 and 170 nmol/l in NZW rabbits (Schiller and McNamara, 1999), between 8 and 16 ng/ml in male and female Japanese rabbits [measured with an IGF-I RIA from Coulter Co. Ltd (Fu et al., 2007)], between 17 and 123 ng/μl in NZW rabbit fetuses of the control groups during days 21 and 31 of gestation [measured with a human IGF-I RIA from Diagnostic Systems Laboratories (Thakur et al., 2000)], and between 245 and 371 ng/ml in 30-day-old male and female NZW rabbits [measured with an IGF-I RIA from the Tianjin Juding Company (Zhang and Li, 2010)]. Taken together, the above cited studies reflect the large degree of heterogeneity in measurement methods, the lack of standardization for measurement of IGF-I in rabbits and the need for a systematic evaluation of physiological IGF-I concentrations in rabbits.

Our current study systematically investigated circulating IGF-I concentrations in rabbits of both sexes, between 1 and 6 months of age and from two genetic strains using a validated IGF-I immunoassay. This data set represents a solid basis for the physiological regulation of IGF-I in rabbits and can, additionally, provide a reference point for future studies measuring IGF-I in rabbits. We have demonstrated that female rabbits of the NZW and CB strains have significantly higher IGF-I concentrations compared with those of age- and strain-matched male rabbits. In our study, the differences between sexes became significant in NZW rabbits aged 3 months and older. Puberty in rabbits is initiated after ~2 months of age (Berger et al., 1982). IGF-I concentrations were highest in 1- and 2-month-old rabbits and declined thereafter in both sexes, being significantly lower at 6 months compared with 1- or 2-month-old rabbits. With respect to age and IGF-I concentrations, the timing in rabbits is similar to that in humans, in which the highest IGF-I concentrations are also found during puberty (Bidlingmaier et al., 2014).

Our data indicate that rabbits, in contrast to rodents (Bielohuby et al., 2011b), are a suitable alternative animal model in which to test novel GH agonists and antagonists because they show a human-like response in changes of IGF-I concentration when treated with recombinant human GH and a GHA. Because the amino acid sequence of secreted GH is highly conserved throughout most mammalian species (including rabbits) (Wallis and Wallis, 1995), it is likely that treatment with GH preparations from other species (e.g. porcine or bovine GH) also increases serum IGF-I concentrations in rabbits. Rabbits could be especially useful to investigate the pharmacodynamics of GHAs because the previously generated bGH-overexpressing rabbit model shares many similarities with the pathophysiological metabolic, histological and anatomical alterations – including acromegalic lesions and insulin resistance – that are frequently seen in individuals with acromegaly (Costa et al., 1998). By contrast, it has been pointed out that GH-overexpressing mouse lines are not a suitable model to study human acromegaly because, although they display a giant phenotype, the typical pathophysiological alterations associated with acromegaly are absent or less pronounced in rodents (Costa et al., 1998). Similarly, GH-overexpressing pigs do not display the sclerotic pathophysiology that is observed in individuals with acromegaly (Pinkert et al., 1994). The acrogemalic phenotype observed in GH-overexpressing rabbits together with the human-like pharmacodynamic properties of IGF-I in rabbits, including their responsiveness to exogenous treatment with recombinant GH and GHAs, renders the rabbit as a valuable research model for this field.

In summary, our experiments have shown that IGF-I concentrations in rabbits display pharmacodynamic behaviors similar to those in human in response to treatment with exogenous recombinant human GH and a GHA. Because IGF-I is not a suitable pharmacodynamic parameter to monitor the action of exogenous GH in rodents, our results open up the possibility to use rabbits in combination with measurements of IGF-I concentration as a new laboratory animal model to develop new GH agonists. Furthermore, rabbits showed a human-like decline of serum IGF-I levels in response to treatment with the GHA Pegvisomant at doses comparable to those used in the clinic, thus enabling research using rabbits on novel therapeutic options for the treatment of pathological GH excess. Similar to other mammals, including humans, rabbits display variations in IGF-I concentrations depending on sex, age and genetic background. Our data on IGFBPs indicate that IGFBPs also play an important biological role in rabbits, but with species specific differences – IGFBP-2 instead of IGFBP-3 is the most abundant IGFBP in rabbits. Furthermore, IGFBP-2 is a good marker for treatment with exogenous recombinant human GH, but not for GHA administration. Second, our assay validation experiments together with the LC-MS/MS data have shown that the human IDS-iSYS automated IGF-I assay can serve as a reliable platform for high-throughput analyses of IGF-I in rabbit serum. The true IGF-I concentrations are approximately twofold higher than the results obtained using the immunoassay, most likely because of a lower binding affinity of the labeled antibody in this human IGF-I immunoassay. By using this automated system, translational research can be facilitated and this might aid development of novel therapeutics to treat pathological conditions of the GH-IGF system.

## MATERIALS AND METHODS

### Validation of the iSYS IGF-I immunoassay for measurement of rabbit IGF-I

#### Serum samples and reagents

The validation of the IDS-iSYS IGF-I immunoassay (IDS, Boldon, UK) for measurement of rabbit IGF-I was performed using commercially available rabbit serum samples (Charles-River, Sulzfeld, Germany). Following sample delivery on dry ice, several aliquots were immediately prepared and stored at −80°C until analysis. For the analysis of matrix effects on the automated system, rabbit serum samples and ten random native human serum samples, which remained after routine diagnostic analyses (‘remnant’), were used. All human serum samples derived from a study which had been approved by the respective local institutional review board, and informed consent was obtained from participants. The source of recombinant human IGF-I (10 mg/ml) was Increlex^®^ (Ipsen, Ettlingen, Germany). Custom-made recombinant rabbit IGF-I (0.5 mg/ml) was prepared by Protein Laboratories Rehovot (PLR, Rehovot, Israel). The recombinant rabbit IGF-I protein was overexpressed in *Escherichia coli* as insoluble protein, refolded and purified to homogeneity as a monomeric protein by using anion-exchange chromatography followed by size exclusion chromatography (analytical purity >95%; monomer content >90%). Initially, the recombinant rabbit IGF-I concentration was determined by reading the absorbance at 280 nm and employing the computer programs of DNAman and/or the PC GENE computer analysis program of protein sequences (IntelliGenetics, Hilton Head, SC, USA). Recombinant rabbit IGF-I is biologically active when compared to human IGF-I. The 50% effective dose (ED_50_), calculated by the dose-dependent proliferation of human MCF7 cells is 5 to 25 ng/ml in the cell culture mixture, depending on culture conditions. Its activity is 30–40% compared to that of human IGF-I. A single production batch of the recombinant rabbit IGF-I was used for all analyses. When preparing the working solution for the recovery experiments using recombinant rabbit IGF-I, all recombinant rabbit IGF-I concentrations (based on the manufacturer’s data) were independently confirmed by LC-MS/MS analyses, as stated in the relevant Materials and Methods section.

#### Assay validation

All IGF-I measurements were performed on the iSYS IGF-I immunoassay using the supplied reagents and following the manufacturer’s assay instructions [further assay details have been published previously by Bidlingmaier et al. (Bidlingmaier et al., 2014)]. A validation of assay precision, sensitivity, linearity and recovery in rabbit serum was performed according to standard recommendations. For the analysis of the intra-assay precision, ten repeated measurements of IGF-I in six native rabbit sera displaying low, medium and high IGF-I concentrations were performed. The precision in the low range was determined using an additional five native rabbit samples with previously measured (i.e. known) IGF-I concentrations. These five samples were divided into four aliquots each and diluted with assay buffer [containing NaCl, Tris-aminomethane, NaN_3_, Tween-40, BSA:BSA, bovine γ-globulin and diethylenetriaminepetaacetic acid (DTPA)] to obtain samples to yield expected IGF-I concentrations between 20 and 25, 15 and 20, 10 and 15, and 5 and 10 ng/ml. Measurement of IGF-I was repeated ten times in each diluted sample, to obtain a total of 200 IGF-I measurements. Mean coefficients of variation from these ten measurements were calculated. The inter-assay variability was investigated in six native rabbit samples (low, medium and high IGF-I concentrations, singlicate measurements) in which IGF-I concentrations were measured over five different assay runs (on five measurement days). Dilution linearity was tested in two low and high rabbit sera (serum A and B, see Table 2) and in two low and high human samples (serum A and B, see Table 2) with IGF-I concentrations between 15 and 585 ng/ml (rabbit) and 12–527 ng/ml (human). IGF-I concentrations were then measured in native rabbit and human samples, and in samples which had been diluted serially 1 in 2 with assay buffer. In a second experiment, three random native rabbit samples were serially diluted with assay buffer and measured in relation to serial dilutions of the reference materials (recombinant rabbit IGF-I and recombinant human IGF-I). For the dilution of the reference material and generation of the standard curves, serial dilutions of recombinant rabbit IGF-I and recombinant human IGF-I dissolved in assay buffer were used (range: recombinant rabbit IGF-I, 8–370 ng/ml; recombinant human IGF-I, 19–1027 ng/ml). For the analysis of recovery, aliquots with the indicated amounts of recombinant rabbit IGF-I were prepared (dissolved in assay buffer) and measured. The ratio of the observed over the expected concentrations (i.e. recovery) is displayed as a percentage in Table 3. Recovery was also investigated by using recombinant human IGF-I (dissolved in assay buffer) and by spiking human and rabbit serum samples with recombinant rabbit IGF-I.

### Analysis of IGF-I by using LC-MS/MS

In a subset of rabbit samples, IGF-I was also measured by using LC-MS/MS as described in detail recently (Cox et al., 2014). Furthermore, concentrations of recombinant rabbit IGF-I in the working aliquots for the recovery experiments were independently confirmed by using LC-MS/MS analyses to yield expected IGF-I concentrations between 12.6 and 3780 ng/ml.

### Analysis of IGFBPs by western ligand blotting in rabbit serum

IGFBPs were analyzed in all sera of rabbits from different genetic backgrounds, age groups and both genders, as well as in a subset of samples from the recombinant human GH and GHA experiments (samples from time points 0, 24, 72 and 168 h after the first injection with the indicated substance) by using quantitative western ligand blot analysis, as described previously (Hossenlopp et al., 1986; Laeger et al., 2014). Briefly, serum samples and serial dilutions of recombinant human IGFBP standards (R&D Systems, Wiesbaden, Germany) were diluted 1 in 20, boiled in sample buffer [312.5 mM Tris (pH 6.8), 50% (w/v) glycerol, 5 mM EDTA (pH 8), 1% (w/v) SDS and 0.02% bromophenol blue] for 5 minutes. Proteins were separated by using SDS-PAGE followed by the transfer onto a polyvinylidene fluoride membrane (Millipore, Bedford, USA). The blots were blocked and then incubated with biotin-labeled human IGF-II (1 in 500; BioIGF2-10; ibt-systems, Binzwangen, Germany). The binding proteins were detected by using enhanced chemiluminescence using LuminataTM Forte (Millipore, Bedford, USA). Bands were visualized on a KODAK Image Station 4000MM (Molecular Imaging Systems, Carestream Health, New Haven, USA) and quantified by using ImageQuant 5.2 software (Molecular Dynamics). Signal intensities were corrected for background and quantified by using human recombinant standards as calibrators (R&D Systems, Wiesbaden-Nordenstadt, Germany) on each blot. Curve fitting was performed by using a four parametric nonlinear regression (HILL equation) of each separate IGFBP. The calculation of the IGFBP concentrations in serum was performed using the software GraphPad Prism4 and corrected for dilution and volume per lane of each sample. The inter-assay coefficients of variation for IGFBP-3, IGFBP-2 and IGFBP-4 were between 6% and 21%.

### Analysis of biological variability in rabbit samples

For the analysis of biological variability of IGF-I concentrations in rabbits, IGF-I concentrations were measured in the serum of male and female rabbits from two different strains of (NZW and CB) rabbit (13-weeks-old, *n*=7 male and *n*=8 female for each strain). Furthermore, IGF-I was measured in serum samples of male and female NZW rabbits [Crl:KLB(NZW)] which were 1, 2, 3, 4 and 6 months old (*n*=10 per sex and age group). In addition, serum IGF-I concentrations were determined in 30 serum samples of 1- and 6-month-old male and female NZW rabbits by using LC-MS/MS analysis, as recently described (Cox et al., 2014). All native serum samples were purchased from an animal supplier (Charles-River, Sulzfeld, Germany). Rabbits were housed in the company’s building (Charles-River) and blood samples were custom-collected from the rabbits’ ear veins for this purpose by skilled company staff members. Following sample delivery on dry ice, several aliquots were immediately prepared and stored at −80°C until analysis.

### Effects of treatment with GH and a GHA on intact rabbits

All experiments involving the treatment of rabbits with GH and a GHA were conducted at the University of Isfahan, Isfahan, Iran. For this purpose, male NZW rabbits aged 30 weeks with an initial body weight of 2100±266 g (mean ± s.d.) were purchased from the Pasteur Institute (Tehran, Iran). Two rabbits were housed per cage at a temperature of 23±2°C and a relative humidity of 45±10%. All animals were allowed free access to water and a commercial rabbit pellet diet supplied by the Pasteur Institute. All animal experiments were approved by the local ethical committee of the University of Isfahan.

Rabbits were randomly divided into three groups seven days before the start of the study. Rabbits received subcutaneous injections of either recombinant human growth hormone (recombinant human GH, Norditropin; Novo Nordisk), growth hormone antagonist Pegvisomant (Somavert, Pfizer) or phosphate buffered saline, PBS (CMG, Iran) at 9 am every day, or as indicated in the protocol (for multiple injections). The Norditropin and Somavert solutions were prepared according to the manufacturer’s recommendations and further dilutions were made, if necessary, using PBS to obtain the injection concentration of 2.5 mg/ml. Rabbits were injected with 1 mg per kg of body weight of the relevant drug or with equal volumes of PBS (recombinant human GH, *n*=6 per group; GHA, *n*=10 per group). Blood samples were taken from the ear veins before administration of drugs or at the specified time points. Injections of recombinant human GH were performed directly after sampling the baseline blood sample (0 h) and 48 or 56 h after the first injection. Blood samples for the experiments involving recombinant human GH were collected at baseline, as well as 4, 8, 24, 48, 56, 72 h after the baseline sample. For the experiments involving Pegvisomant, rabbits were injected with this GHA after collecting the baseline sample (0 h) and at six additional time points in 24 h intervals (after 24, 48, 72, 96, 120 and 144 h). Blood samples for these experiments were collected at baseline, as well as 24, 48, 72, 96, 120, 144, 168 h after the baseline sample. Blood samples were allowed to clot at room temperature for 1 h, centrifuged and stored at −80°C before shipment on dry ice to be assayed. Body weight measurements were performed using a digital scale (Sartorius) before injection or blood sampling. Serum samples were then used to measure circulating IGF-I with the automated iSYS immunoassay as described above. Furthermore, circulating concentrations of Pegvisomant or recombinant human GH were analyzed in rabbit samples using a time-resolved fluorescence assay. Detailed descriptions of this method have been published elsewhere (Veldhuis et al., 2002; Veldhuis et al., 2010).

### Statistical analysis

Statistical analysis was performed using the SPSS software package (SPSS, version 15.0, Chicago, USA), Microsoft Excel and GraphPad Prism (GraphPad Software, Version 5, La Jolla, USA). Non-parametric Mann–Whitney U tests were used for statistical comparison between two groups (e.g. IGF-I concentrations in male vs. female rabbits, Fig. 2) whereas one-way (Fig. 5) and two-way ANOVA with subsequent Bonferroni correction for multiple-comparison post-hoc tests was used for comparisons between three or more groups (e.g. comparison of IGF-I concentrations in different age groups or the effects of treatment with Pegvisomant on IGF-I; Figs. 3, 4). Correlation analyses for the comparison of measurements methods (immunoassay versus LC-MS/MS) and for the correlation analyses of IGFBPs with serum IGF-I were performed by non-parametric Spearman’s analysis and by Passing–Bablok regression. The Passing–Bablok comparison was used to compare the two measurement methods (immunoassay versus LC-MS/MS) because this non-parametric method does not require a normal distribution of the data and is very robust against the distribution of uncertainty between the two methods and individual outliers. The cut-off for statistical significance was set at *P*<0.05. Unless stated differently, all data are presented as means ± standard error of the mean (s.e.m.).

## Supplementary Material

Supplementary Material
