# Supplementary material for: Validation of serum IGF-I as a biomarker to monitor the bioactivity of exogenous growth hormone agonists and antagonists in rabbits
Source: Dis Model Mech. 2014 Sep 19;7(11):1263–73. doi: 10.1242/dmm.016519 (PMC4213730; doi:10.1242/dmm.016519)
Supplement: Supplementary Material [file supp_7_11_1263__index.html]

Validation of serum IGF-I as a biomarker to monitor the bioactivity of exogenous growth hormone agonists and antagonists in rabbits — Supplementary Material 

# Validation of serum IGF-I as a biomarker to monitor the bioactivity of exogenous growth hormone agonists and antagonists in rabbits

## DMM016519 Supplementary Material

**Files in this Data Supplement:**

- **Supplementary Material**
