# Supplementary material for: Validation of serum IGF-I as a biomarker to monitor the bioactivity of exogenous growth hormone agonists and antagonists in rabbits
Source: Dis Model Mech. 2014 Sep 19;7(11):1263–73. doi: 10.1242/dmm.016519 (PMC4213730; doi:10.1242/dmm.016519)

Supplementary table and figure legends

Supplementary Table 1

Supplemental table 1 shows the within-run precision (intra-assay variability) for the iSYS immunoassay for 10-fold measurement of IGF-I in 6 native rabbit samples displaying either low, medium or high IGF-I concentrations. Mean within-run precision amounted to 1.1 – 3.3% CV (168 – 478 ng/ml).

Supplementary Table 2

Supplemental table 2 shows the total precision (inter-assay variability) for the iSYS immunoassay for measurement of IGF-I in 6 native rabbit samples at 5 different measurement days. The automated assay was freshly calibrated on each measurement day. Mean (total) precision amounted to 11.7 – 3.3% CV (173 – 396 ng/ml).

Supplementary Table 3

Supplemental table 3 shows the precision in the low range (Limit of Quantitation (LoQ)) for the iSYS immunoassay for measurement of IGF-I in rabbit serum. 5 native rabbit sera with previously measured (i.e. known) IGF-I concentrations were used. These 5 samples were divided into 4 aliquots each and diluted with assay-buffer to obtain samples yielding expected IGF-I concentrations between 20-25, 15-20, 10-15 and 5-10 ng/ml, respectively. Measurement of IGF-I was repeated 10 times in each diluted sample, for a total of 200 IGF-I measurements. Mean coefficients of variation (CV) from these 10 measurements were calculated for each IGF-I concentration interval. The analyses confirmed a functional sensitivity with an acceptable mean CV of the assay for rabbit IGF-I below 10 ng/ml (7.3ng/ml).

Supplementary Figure 1

Supplemental figure 1 displays circulating concentrations of the growth hormone antagonist Pegvisomant (GHA, blue) and of recombinant human growth hormone (rhGH, red) in rabbits after a single injection with 1 mg/kg of the respective drug. Serum samples have been collected prior to injection at baseline ("0h") and 1, 2, 4, 8, 24 and 48h after injection with the respective drug. The pharmacokinetic behaviour of GHA and rhGH was clearly different in rabbits: rhGH concentrations peaked 1h after the injection, the highest concentrations of GHA were measured 8h after the injection. As expected, rhGH and GHA were not detectable

in PBS treated controls. For illustrative purposes in the figure, a black line (slightly above zero) shows the concentrations in controls. Data are presented as means±SEM (n=3/treatment group).

Table S1

Intra-assay variability

	**low 1**	**low 2**	**med 1**	**med 2**	**high 1**	**high 2**
**replicate**	**sample 1**	**sample 2**	**sample 3**	**sample 4**	**sample 5**	**sample 6**
**1**	163	195	277	263	429	472
**2**	167	203	287	268	432	485
**3**	160	203	295	274	433	480
**4**	161	199	276	260	421	472
**5**	171	205	288	268	429	475
**6**	174	201	288	267	427	480
**7**	167	195	275	262	410	475
**8**	175	198	280	276	432	483
**9**	174	199	278	270	423	485
**10**	169	201	281	260	414	471
**Mean [ng/ml]**	**168.1**	**200**	**282.4**	**266.9**	**425.0**	**477.8**
**SD**	**5.5**	**3.5**	**6.7**	**5.7**	**7.8**	**5.4**
**% CV**	**3.3**	**1.8**	**2.4**	**2.1**	**1.8**	**1.1**

Table S2

Inter-assay variability

Sample	Measurement 1	Measurement 2	Measurement 3	Measurement 4	Measurement 5	Mean [ng/ml]	SD	% CV
1	182	176	176	180	175	**178**	**3**	**1.7**
2	166	169	179	179	172	**173**	**6**	**3.3**
3	183	176	174	172	174	**176**	**4**	**2.4**
4	401	394	387	408	389	**396**	**9**	**2.2**
5	352	344	357	370	343	**353**	**11**	**3.1**
6	352	356	368	365	355	**359**	**7**	**1.9**

Table S3

Precision in the low range

Samples	Mean (and range of means) measured IGF-I concentrations from 10 measurements in 5 different rabbit sera [ng/ml]	Mean CV from 10 measurements in 5 different rabbit sera [%]
1-5	23.8 (23.1-24.6)	5.4 (2.6-8.8)
6-10	18.0 (17.7-18.5)	5.4 (3.1-7.6)
11-15	13.0 (12.9-13.2)	5.1 (3.7-7.5)
16-20	7.3 (7.2-7.7)	7.9 (4.9-10)

Supplementary Figure 1

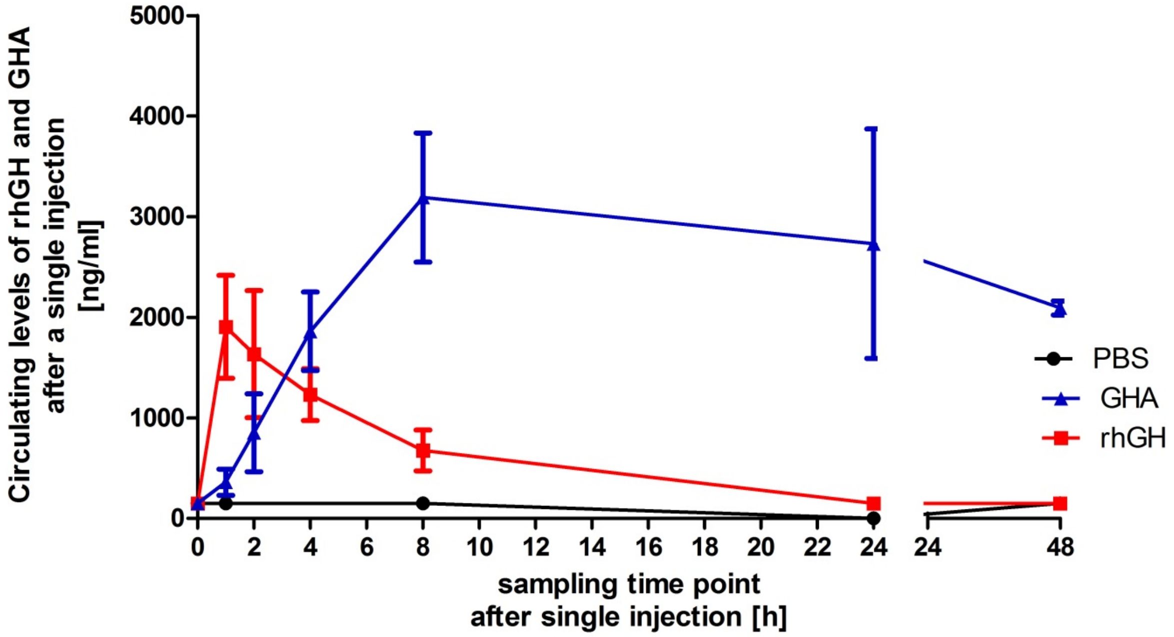

Supplement: Supplementary Material [file supp_7.11.1263_DMM016519.pdf]
